# The proteome of the outer membrane vesicles of an Antarctic bacterium *Pseudomonas syringae* Lz4W

**DOI:** 10.1016/j.dib.2015.07.001

**Published:** 2015-07-11

**Authors:** Heramb.M. kulkarni, Ch. V.B. Swamy, Medicharla.V. Jagannadham

**Affiliations:** CSIR-Centre for Cellular and Molecular Biology, Uppal Road, Tarnaka, Hyderabad 500007, India

**Keywords:** Outer membrane vesicles, Gram-negative bacteria, Physiology, Proteomics, ESI-MS/MS

## Abstract

Outer membrane vesicles (OMVs) of gram-negative bacteria are released during all growth phases and play an important role in bacterial physiology. They consist of lipids, proteins, lipopolysaccharides and other molecules. The OMVs of the Antarctic bacterium *Pseudomonas syringae* Lz 4W were isolated and identified their proteins. The mass spectral data set deposited with PRIDE, accession number PXD 000221 is presented in this report. The proteins identified from the OMVs of *P. syringae* Lz4W, data of this study were published in the Journal of proteome research [1].

Specifications Table

**Table:** 

Subject area	*Biology,Chemistry*

More specific subject area	*Microbial Proteomics, Bacterial physiology*
Type of data	*LC-ESI-MS/MS data*
How data was acquired	*Mass spectrometry*
Data format	*MS/MS data obtained from Orbitrap velos (Thermo scientific) was used for the identification of proteins.*
Experimental factors	*The protein of OMVs isolated form an Antarctic bacterium was identified using SEQUEST, MASCOT and PEAKS programs. The proteins commonly identified in all the three programs were accepted for further functional analysis.*
Experimental features	*In gel digested proteins with Trypsin were extracted with* 5*% TFA in* 50*% acetonitrile. Dried and desalted the peptides with Zip-tip C*18 *columns and loaded on Nano LC-ESI MS/MS.*
Data source location	*CSIR-Centre for Cellular and Molecular Biology, Hyderabad, India.*
Data accessibility	*Achieves of PRIDE, PXD* 000221. The identified proteins were published in Journal of Proteome research [1].

Value of the data

• The proteins associated with OMVs of the Antarctic bacterium *P. syringae* Lz4W were identified to understand their structure and functions.
• Outer membrane, periplasm proteins were present as major components of OMVs.
• More than 500 proteins were identified using nano LC-ESI-MS/MS.

## 1 Data, experimental design, materials and methods

### 1.1 Bacterial strain growth conditions

The bacterium *P. syringae* Lz4W was isolated from soil samples collected in and around Lake Zub, Schirmacher Oasis, Antarctica. The geographical coordinates of the sampling area are 70°45′12″S and 11°46′E. This bacterial strain can grow between 0 °C and 30 °C with an optimum growth temperature of 22 °C [2]. It is a well studied bacterium in our laboratory whose genome sequence was recently published [3]. The bacterium *P. syringae* Lz4W was grown at 22 °C in Antarctic bacterial Medium (ABM) that contains 0.5% peptone and 0.2% yeast extract with aeration (by continuous shaking up to late stationary phase (OD 600 nm=1.1–1.2) for the preparation of OMVs.

### 1.2 Preparation of OMVs

The OMVs were prepared from bacteria cultures by using the method described in literature [4,5]. with minor modifications. In brief, the cultures of all bacterial strains were grown up to stationary phase in respective growth conditions. The cells were separated from the culture by centrifugation at 10,000 g for 10 min. at 4 °C. The further steps of purifications involved filtration of the supernatant, ultracentrifugation and density gradient ultracentrifugation. The supernatant was filtered by using 0.45 μm membrane (Millipore). The OMVs in the filtrate were pelleted by ultracentrifugation at RCFmax of 150,264 g (36,000 rpm for ‘type 45 Ti’ rotor, Beckman) for 3 h at 4 °C in Beckman ultracentrifuge in polycarbonate tubes. The pellet of OMVs was obtained which was subsequently re-suspended in 10 mM phosphate buffer saline (pH 7.4). This preparation of OMVs was further purified by sucrose density gradient centrifugation. In polyallomer tubes, equal layers of sucrose solutions (prepared in 10 mM phosphate buffer saline pH 7.4), 70%, 60%, and 20% were added from the bottom to top. The suspension of OMVs was layered on the top of it. The tubes were ultracentrifuged at RCFmax of 164,609 g (35,000 rpm for ‘SW 60 Ti’ rotor, Beckman) at 4 °C for 6 h. All the fractions were collected from the gradient. Aliquot from each of them was diluted 25 times. They all were tested for the presence of OMVs by using dynamic light scattering (DLS). The fractions that were detected to contain OMVs were pooled together, diluted to 4 mL and subjected to ultracentrifuge at RCFmax of 163,202 g (55,000 rpm for ‘TLA-100.3’ rotor, Beckman) at 4 °C for 2 h. The pellet of purified OMVs was resuspended in 10 mM phosphate buffer (pH 7.4) for OMVs of *P. syringae* Lz4W and in 10 mM phosphate buffer saline (pH 7.4), and was stored at −20 °C until used for experiments.

### 1.3 Dynamic light scattering

The size distribution of the OMVs was monitored by using DLS. The preparations of OMVs were diluted with phosphate buffer (10 mM, pH 7.4) to a protein concentration of 0.04–0.06 µg/mL and were subjected to measure the size distribution. The scatter of the diluted OMVs from *P. syringae* Lz4W was recorded by Photocor instrument (College Park, MD, USA) at 90° angle at 22 °C with a laser of wavelength 632 nm. The data was analyzed by Dynals software to obtain the average hydrodynamic radius of the OMVs.

### 1.4 Transmission electron microscopy (TEM)

The OMVs were visualized by using TEM using a Jeol Transmission Electron Microscope (JEM 2100, Tokyo, Japan) at 200 kV. The sample was taken on a carbon coated grid and negatively stained with uranyl acetate for observing the vesicles. Then it was transferred to TEM with cryotransfer holder and maintained there at −175 °C. Images were recorded in TEM (from Jeol) at 200 kV. For all experiments the images were captured, visualized and stored by Gatan Camera.

### 1.5 Quantification of OMVs

The yield of OMVs was quantified per CFU of the culture by using protein and lipid based assays as described earlier [6]. The protein content of the preparation was determined by Bradford method using Biorad protein estimation kit and the lipid content was measured by fluorescence assay using a lipophilic dye FM4-64. For lipid estimation the modification described by Frias et al. (2010) [7] was used. The standard curve of the lipids was prepared by using small unilamellar vesicles (SUVs) prepared from phosphatidylglycerol (POPG). Different concentrations of SUVs of POPG were added with 7 µg/mL of final concentration of FM4-64. After incubating the samples for 10 min. at 37 °C the samples were subjected to the excitation at 515 nm and the emission at 635 nm was measured on F-7000 fluorescence spectrometer (Hitachi, Tokyo, Japan). The excitation and emission slits were set at 5 nm each. The OMVs were diluted and the fluorescence measured in similar way. The CFUs/mL of the culture at the time of harvest was decided by diluting it to 10^10^ and spreading onto ABM agar plates in triplicate. The plates were subsequently incubated at 24 °C for 30 h to detect bacterial contamination.

### 1.6 SDS-PAGE profiles of OMVs

The OMVs were added with 4 times of chilled acetone (v/v) to precipitate their proteins. The obtained proteins were then separated on 12% SDS-PAGE gels at constant current of 25 mA and the bands were visualized by using coomassai blue [8]. The protein profiles of OMVs thus obtained were captured by a gel-doc system (BioRad).

### 1.7 Mass spectrometry of digested proteins

The tryptic peptides were subjected to mass spectrometry using LC-ESI-MS/MS Orbitrap velos instrument obtained from Thermo Scientific (San Jose, CA). The peptides were separated using Proxeon LC system on a Biobasic C18 (100 mm×0.18 mm) reverse phase nano column, with a pore size of 300 Å and particle size of 5 μm (New Objectives, MA, USA) on a 90 min gradient. The LC system was connected to ESI-MS/MS which recorded the CID MS of the peptides. The flow rate was set at 350 nL/min. The mobile phases A and B were 0.2% formic acid in water and 0.2% formic acid in 95% ACN respectively. The gradient was started at 10 min and increased to 60% B in 40 min and to 100% B in 55 min. The MS and MS/MS spectra were obtained at a heated capillary temperature of 200 °C and the ESI voltage was set at 1.6 kV. The peptides were fragmented using normalized collision energy of 35%. The MS/MS spectrum of the top 20 peptides with a signal threshold of 500 counts was acquired with 10 ms activation time and a repeat duration of 30 s.

### 1.8 Identification of proteins

The proteins from the Antarctic bacterium were identified earlier using different strategies, when the genome sequence is not available [9–12]. Recently, the draft genome sequence of the Antarctic bacterium *P. syringae* Lz4W shows that it contains 4450 entries and deposited at DDBJ/EMBL/GenBank under the accession number AOGS00000000 [3]. The MS/MS spectra of the multiply charged peptides were searched against this database of *Pseudomonas* Lz4W. The LC-ESI MS/MS spectra were analyzed using the Proteome Discoverer Version 1.4 supplied by the manufacturer. All the MS/MS spectra were analyzed using SEQUEST (Thermo fisher scientific) and MASCOT selecting the enzyme trypsin and applying the search parameters of precursor tolerance of 10 ppm and a fragment tolerance of 0.8 Da. The increase in mass due to the oxidation of methionine (15.99 Da) and carboxyamidomethylation of cysteine (57.02 Da) were set as variable and fixed modification respectively. Only peptides identified with high confidence were included in the list. All the proteins were identified by at least two unique peptides. Proteins were also identified by Peaks 6 software (Peaks DB), by using the same search parameters. Peaks algorithm determines the *de novo* sequencing of the peptides from the MS/MS spectra, and these sequences are matched against the *P. syringae* Lz4W database for identification of the proteins. Average Local Confidence (ALC) score set for acceptance was at 30 for *de novo* sequences. Since the Total Local Confidence depends upon the length of individual peptide, it was not considered for setting any cut-off. The −10lgP score was set at 50 for accepting the match from the database search. The probability of a wrong hit at this score is≤0.001%. The proteomics data have been deposited to the ProteomeXchange consortium with identifier PXD000221 (http://proteomecentral.proteomexchange.org) via the PRIDE partner repository [13]. The proteins identified by different algorithms SEQUEST, MASCOT, PEAKS and were combined; proteins common to all three programs were accepted as confident hits and used for further analysis.
